# Seroprevalence of SARS-CoV-2 antibodies during the third wave of COVID-19 in the Seoul metropolitan area of Korea

**DOI:** 10.4178/epih.e2022085

**Published:** 2022-09-30

**Authors:** Kyuhyun Yoon, Jayeun Kim, Kyong Ran Peck, Hyun Soo Kim, Hyukmin Lee, Yoo-Sung Hwang, Soon Young Lee, Sung-il Cho, Hun Jae Lee, Yeong-gyeong Kim, Byoungguk Kim, June-Woo Lee, Ah-Ra Kim, Hyeon Nam Do, Dong-Hyun Kim

**Affiliations:** 1 Institute of Health and Environment, Seoul National University, Seoul, Korea; 2 Korea Institute of Child Care and Education, Seoul, Korea; 3 Division of Infectious Diseases, Samsung Medical Center, Sungkyunkwan University School of Medicine, Seoul, Korea; 4 Department of Laboratory Medicine, Hallym University Dongtan Sacred Heart Hospital, Hallym University College of Medicine, Hwaseong, Korea; 5 Department of Laboratory Medicine, Severance Hospital, Yonsei University College of Medicine, Seoul, Korea; 6 Seegene Medical Foundation, Seoul, Korea; 7 Department of Preventive Medicine and Public Health, Ajou University School of Medicine, Suwon, Korea; 8 Department of Public Health Science, Graduate School of Public Health, Seoul National University, Seoul, Korea; 9 Department of Social and Preventive Medicine, Inha University College of Medicine, Incheon, Korea; 10 Division of Vaccine Clinical Research Center for Vaccine Research, National Institute of Infectious Diseases, National Institute of Health (NIH), Korea Disease Control and Prevention Agency (KDCA), Cheongju, Korea; 11 Department of Social and Preventive Medicine, Hallym University College of Medicine, Chuncheon, Korea; 12 Institute of Social Medicine, Hallym University, Chuncheon, Korea

**Keywords:** Seroepidemiologic studies, COVID-19 antibody testing, Cumulative incidence, Asymptomatic states

## Abstract

**OBJECTIVES:**

After the third wave of coronavirus disease 2019 (COVID-19), by mid-February 2021, approximately 0.16% of the Korean population was confirmed positive, which appeared to be among the lowest rates worldwide at that time. However, asymptomatic transmission is challenging for COVID-19 surveillance. Therefore, a community-based serosurvey of severe acute respiratory syndrome coronavirus 2 (SARS-CoV-2) infection was conducted to understand the effectiveness of Korea’s strong containment strategy.

**METHODS:**

We collected 5,002 residual sera samples from January 30 to March 3, 2021, from 265 medical facilities in Seoul, 346 in Gyeonggi Province, and 57 in Incheon. Sixty samples from tertiary institutions were excluded. We defined the sub-regions according to the addresses of the medical facilities where the specimens were collected. Elecsys Anti-SARS-CoV-2 was used for screening, and positivity was confirmed using the SARS-CoV-2 sVNT Kit. Prevalence was estimated using sampling weights and the Wilson score interval for a binomial proportion with a 95% confidence interval.

**RESULTS:**

Among the 4,942 specimens, 32 and 25 tested positive for COVID-19 in the screening and confirmatory tests, respectively. The overall crude prevalence of SARS-CoV-2 antibodies was 0.51%. The population-adjusted overall prevalence was 0.55% in women and 0.38% in men. The region-specific estimation was 0.67% and 0.30% in Gyeonggi Province and Seoul, respectively. No positive cases were detected in Incheon.

**CONCLUSIONS:**

The proportion of undetected cases in Korea remained low as of early 2021. Therefore, an infection control strategy with exhaustive tracing and widespread pre-emptive testing appears to be effective in containing community spread of COVID-19.

## INTRODUCTION

**Figure 1.**
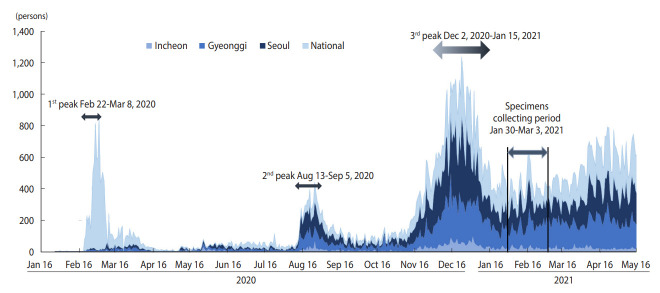
Trends in daily new cases in the capital area compared to national and study period. The national value is the total. The capital area’s total is sum of each value of 3 regions: Incheon, Gyeonggi Province, and Seoul. Serum antibody testing was performed 2 weeks after January 16, when the third peak began to subside.

Since late 2019, an unprecedented pandemic crisis caused by severe acute respiratory syndrome coronavirus 2 (SARS-CoV-2) has had devastating effects on global health and the economy. As of October 2021, > 200 million people have been infected with the virus and approximately 5 million people have died worldwide [1]. In addition, there have been various reports on newly emerging variants of SARS-CoV-2, including Beta and Omicron [2,3]. Korea’s response strategy to coronavirus disease 2019 (COVID-19) during the first 2 years of this pandemic was defined as 3T: massive testing, aggressive tracing, rapid isolation, and efficient treatment. This strategy was found to be effective in containing the spread of COVID-19 in the community [4]. At the end of 2020, a third peak occurred in Seoul, the capital of Korea (Figure 1). From that period, the proportion of confirmed cases in which the epidemiological route of transmission was not identified at the time of confirmation increased; 12.9% of confirmed cases for the week of November 15, 2020 were labeled “under investigation,” increasing to 27.95% for the week of December 13, 2020 [5]. This finding suggests that there were many undetected cases in the community.

Unlike SARS and Middle East respiratory syndrome, as previous 21st-century epidemic diseases caused by coronaviruses, some cases of COVID-19 are completely asymptomatic [6]. Transmission through asymptomatic infected individuals was outside the scope of available epidemiological tracing techniques, resulting in an increase in the number of undetected cases in the community [6-8]. Therefore, it is important to determine the overall transmission pattern of this virus through community seroprevalence studies.

In a meta-analysis performed across 968 seroprevalence studies in 2020 [9], the median value of seroprevalence differed by region, from 0.6% in Southeast Asia, East Asia, and Oceania to 19.5% in sub-Saharan Africa. Nationwide studies have also shown a lower seroprevalence than regional or local population-based studies. People aged 18-64 years showed higher seroprevalence than those aged ≥ 65 years (prevalence ratio, 1.27; 95% confidence interval [CI], 1.11 to 1.45).

The Korea Disease Control and Prevention Agency (KDCA, formerly Korea Centers for Disease Control and Prevention) confirmed only 5 positive antibody tests in 5,284 people who participated in the Korea National Health and Nutrition Examination Survey, demonstrating a very low prevalence of 0.09% [10]. A few studies have been conducted on the prevalence of antibodies against COVID-19 using secondary blood samples collected for medical examinations or other diagnostic tests in Korea [11-13]. The seroprevalence in residual blood sera (1,500 samples) obtained from visitors to medical institutions in 5 districts in southwestern Seoul was 0.07% [13].

More than 70% of the national confirmed cases during the third wave at the end of 2020 were from the capital areas (Seoul, Gyeonggi Province, and Incheon) [5], where > 50% of the total population of Korea resides. The steady number of cases in this area warrants a critical evaluation of Korea’s quarantine policy and response to COVID-19.

This study aimed to estimate the prevalence of antibodies against SARS-CoV-2 in densely populated metropolitan areas in Korea to reflect the potential risk of undetected infections due to the third peak of COVID-19. The results are expected to show the comprehensive effectiveness of the containment strategies adopted in Korea and help to establish a seroprevalence surveillance system and an evidence-based preparation strategy to respond to new waves.

## MATERIALS AND METHODS

### Survey design and subjects

This survey targeted adults aged ≥ 19 years residing in the capital areas (Seoul, Gyeonggi Province, and Incheon) who underwent blood testing through the Seegene Medical Foundation laboratory. Specimens for the SARS-CoV-2 antibody test were collected from a convenience sample of residual sera. We assumed that asymptomatic community infections would occur in at least 1% of samples. To estimate a binomial proportion, the sample size (*n*) calculated using the Z statistic for 95% confidence, expected prevalence (P), and precision (*d*, effect size) is commonly used (1) [14]:


(1) $$n=\frac{Z^{2}P(1-P)}{d^{2}}$$

To calculate the sample size of a prevalence study for a disease with a low prevalence of < 10%, the precision can be given as half of P [15]. Therefore, we produced an appropriate sample size for a 1% prevalence of 1,521. We targeted 5,000 tests, more than triple the number of samples calculated, considering budgetary resources.

From the residual sera in the collaborating laboratory, we first extracted samples with information on the patient’s sex and age and the location of the medical facility. From the medical facilities that requested blood tests in the capital area, we filtered out community-based primary clinics (including oriental medicine and dental clinics) and small secondary hospitals. Because we were interested in exploring the community-dwelling population, we excluded facilities beyond the boundaries of residential areas, such as tertiary and university-affiliated hospitals. Long-term care facilities were also excluded because facility residents are not community dwellers. The region was defined according to the location of the medical facility where the blood samples were initially collected.

**Figure 2.**
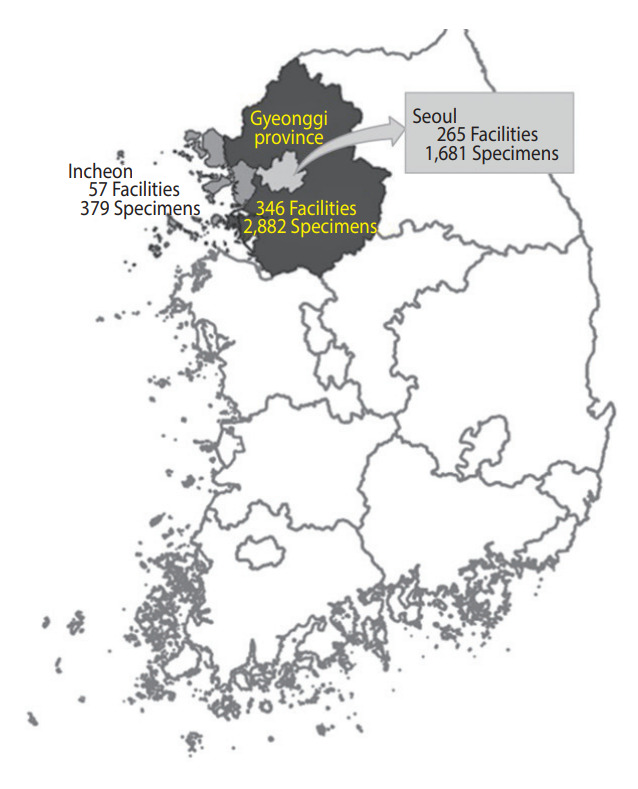
Capital area and residual sera collection information.

Overall, we collected 5,002 residual sera samples from January 30 to March 3, 2021 (Figure 1). After reconfirming the age and sex of the donor and location of the medical facility, 60 samples from tertiary hospitals were excluded. Samples were obtained from a final total of 306 facilities in Seoul, of which 159 (52.0%) were internal medicine. In Gyeonggi Province, samples were obtained from 272 facilities, of which 84 (30.9%) were clinics of general practitioners, 77 (28.3%) were internal medicine, and 35 (12.9%) were hospitals. In Incheon, 37 (39.4%) of the 94 facilities were internal medicine, and 33 (35.1%) were clinics of general practitioners. In addition, facilities practicing obstetrics and gynecology, surgery, and diagnostic medicine were included. We tested 4,942 residual serum samples from these facilities (Figure 2).

### Laboratory tests

As a screening test, residual serum samples were tested using Elecsys Anti-SARS-CoV-2 (Roche, Mannheim, Germany) on a Cobas e 801 analyzer using the electrochemiluminescence immunoassay principle, according to the manufacturer’s protocol. A cut-off index (signal sample/cut-off) of ≥ 1.0 indicated anti-SARS-CoV-2 positivity. The clinical sensitivity and specificity of this test were 99.5% and 99.8%, respectively [16].

As a confirmatory test to decrease false positivity in a low-prevalence setting, we sequentially performed a SARS-CoV-2 surrogate neutralizing antibody test (sVNT Kit, L00847 cPass™ SARS-CoV-2 Neutralization Antibody Detection Kit; GenScript, Piscataway, NJ, USA) according to the manufacturer’s instructions. This simple assay detects antibodies that inhibit the receptor-binding domain–angiotensin-converting enzyme 2 interaction, which is crucial for viral entry into host cells. The percent inhibition of each test was calculated as (1−average optical density [OD] of sample/average OD of negative control) × 100%. A test with a percent inhibition of < 20% or ≥ 20% was considered “negative” or “positive” for SARS-CoV-2 neutralizing antibodies, respectively [17].

A plaque reduction neutralization test (PRNT) was performed prior to screening to determine the reliability of the automated neutralizing antibody test. We used the PRNT results instead of the automated test for 2 specimens that contained insufficient sera for the SARS-CoV-2 sVNT Kit test.

### Statistical analysis

We analyzed the age distribution and calculated the crude prevalence of SARS-CoV-2 antibodies for the sampled populations by dividing both screening-positive cases and confirmed positive cases. To calculate the prevalence of SARS-CoV-2 antibodies, we applied the Wilson score interval for a binomial proportion to estimate the 95% CI. The Wilson interval is recommended as being considerably narrower than the standard interval [18].

To compare the sampled antibody test outcomes in our study and the incidence rate of SARS-CoV-2, we used the nationwide cumulative incidence of SARS-CoV-2, which was reported to the KDCA up to January 16, 2021. We calculated the cumulative incidence rate using the resident registration population from Statistics Korea in February 2021 as the denominator.

All results are presented as prevalence proportions (%) with 95% CIs. The described procedures were conducted using SAS version 9.4 (SAS Institute Inc., Cary, NC, USA).

### Ethics statement

This study was approved by the Public Institutional Review Board Designated by Ministry of Health and Welfare (P01-202102-31-002) and Seegene Medical Foundation Institutional Review Board (SMF-IRB-2021-003).

## RESULTS

Table 1 shows the distribution of the study participants’ age, sex, and region, as well as the overall resident registration population of the region for residents aged ≥ 19 years as of February 2021. The overall age distribution was 47.6± 16.7 (mean± standard deviation). The age and sex distributions of the participants were similar to those of the resident registration population. However, oversampling was observed in Gyeonggi Province and undersampling was observed in Seoul and Incheon.

Participants’ characteristics and SARS-CoV-2 antibody prevalence are shown in Table 2. The total number of positives in the screening and confirmatory tests was 32 and 25, respectively. Positivity on the confirmation test using surrogate neutralizing antibodies was only found in 78% of the positive screening test results. Accordingly, the crude overall prevalence of SARS-CoV-2 antibody was calculated as 0.65% (95% CI, 0.46 to 0.91) and 0.51% (95% CI, 0.34 to 0.75) for screening-based and confirmed positivity, respectively.

The sex-specific prevalence of SARS-CoV-2 antibodies among the screening-positive results was 0.68% (95% CI, 0.43 to 1.09) in females and 0.61% (95% CI, 0.37 to 1.01) in males. If the overall number of positive cases was reduced by analyzing the confirmed neutralizing antibody test, the sex-specific prevalence also decreased to 0.60% (95% CI, 0.37 to 0.99) in females and 0.41% (95% CI, 0.22 to 0.75) in males.

A regional analysis showed that positive cases were only detected in Seoul and Gyeonggi Province, with a higher prevalence observed in Gyeonggi Province (0.87%; 95% CI, 0.59 to 1.28) than in Seoul (0.42%; 95% CI, 0.20 to 0.86).

The estimated SARS-CoV-2 antibody prevalence, adjusting for the population’s sex and age, was 0.60% (95% CI, 0.42 to 0.85) in the screening tests and 0.47% (95% CI, 0.31 to 0.70) in the confirmatory tests in males and females, whereas the cumulative incidence rate using polymerase chain reaction (PCR) of SARS-CoV-2 among the population aged ≥ 19 years was 0.18% in the capital area by January 16, 2021. The estimated prevalence through serological testing was approximately 3 times that of the cumulative incidence through diagnostic testing. Seoul had the highest cumulative incidence (0.24%), followed by Gyeonggi Province and Incheon (0.15 and 0.14%, respectively; Table 2). We calculated these cumulative incidence rates using the number of confirmed cases among residents aged ≥ 19 years in each region, excluding foreigners. Therefore, these values differ from official government statistics.

**Figure 3.**
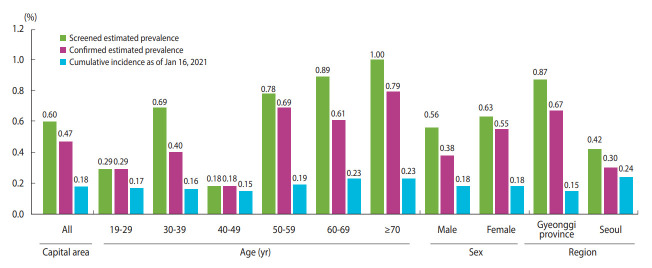
Comparison of seroprevalence and cumulative incidence. The Incheon area, which had zero positive results, is excluded.

Figure 3 shows the differences between the estimated seroprevalence and cumulative incidence rates. Although there was no substantial difference among the cumulative incidence rates by age group, seroprevalence was relatively low in individuals in their 20s and 40s. The cumulative incidence rate was the highest in Seoul, but the serum prevalence was the highest in Gyeonggi Province, approximately twice that in Seoul.

## DISCUSSION

We performed a serological study of the community-dwelling population in the capital area of Korea (Seoul, Gyeonggi Province, and Incheon). A relatively low seroprevalence of 0.60% according to the test using a recombinant protein representing the nucleocapsid (N) antigen and 0.47% according to the test for detection of total neutralizing antibodies, adjusted by sex and age of the population, was confirmed in the capital area, which is the most vulnerable to the spread of SARS-CoV-2 in Korea. When comparing our results with the cumulative incidence rate obtained using PCR-confirmed cases, in mid-January 2021, approximately 2 weeks before our study began, the cumulative incidence rate in individuals aged ≥ 19 years was 0.18%, which is still relatively low compared to that in other countries [5]. The difference between the two values was approximately 0.4%p with the N antigen-antibody test and approximately 0.3%p with the neutralizing antibody test.

Another study in Korea of 4,085 people undergoing medical examinations at health promotion centers in 13 cities across the country between late September and early December 2020 showed a difference of approximately 0.3%p between the seroprevalence and the total incidence [12], which was similar to our results.

However, the antibody prevalence using the rapid antibody test kit, in a study conducted from May 25 to June 5, 2020, in Daegu Metropolitan City, was estimated to be 7.6% (95% CI, 4.3 to 12.2) [19]. The reason for such a high prevalence is presumed to be that the area where the survey was conducted was near the Shincheonji community, where a religious group led the first wave in Korea with a total of 5,214 COVID-19 confirmed cases. Moreover, the accuracy of the immunochromatographic rapid diagnostic kit used may have been a problem. The sensitivity of the test compared to the PCR test results was 100%, but the specificity was only 92%, so the false-positive rate must have been high. The other serological study conducted there during the same period as our study reported a rate of 0.41% (12/2,935), even though the subjects were healthcare workers. The latter finding confirms the limitations of previous studies, while at the same time showing improved reliability [20].

Countries that implemented elimination policies, such as Australia, Taiwan, and Korea, showed lower seroprevalence than countries with mitigation policies, such as the United States and the United Kingdom, regardless of study design and period. Canada showed a different pattern by switching from a mitigation policy to an elimination policy before the situation escalated [21]. Even within the same region, the degree of spread of community infections varied according to each country’s medical delivery system, response, and intensity of sanctions. However, the assessment of responses to the pandemic during the last year using mortality, economic damage, and the stringency of policies showed better effectiveness of the elimination approach for COVID-19 containment [22].

Similar to Korea, the incidence and seroprevalence were particularly low in Australia, where the elimination policy was well applied. In a study of 3,037 asymptomatic patients admitted for planned surgery between June and July 2020 at 11 hospitals in 4 states in Australia, the differences between incidence and seroprevalence were only 0.25% in June 2020 and 0.13% in July 2020 [23]. A study conducted in Sydney between April and June 2020 using blood samples from patients who underwent diagnostic tests, pregnant females who underwent prenatal testing, and plasma donors described seroprevalence differences between antenatal care attendees (0.79%; 95% CI, 0.04 to 0.41), diagnostic pathology service examinees (0.24%; 95% CI, 0.04 to 0.80), and plasmapheresis donors (0.69%; 95% CI, 0.04 to 1.59) [24]. In New South Wales, the total incidence rate on May 31 was approximately 0.04% [25], and the difference from the seroprevalence in the study was still < 1%.

Our study has 3 limitations. First, convenience sampling using residual blood collected from medical facilities, as was done in this study, was not conclusive in terms of regional representation and generalizability, although a recent meta-analysis showed that many studies are still being conducted in hospital settings worldwide due to practical feasibility issues of study performance [26]. Second, owing to the lack of other epidemiological information, it is not known whether the positive results were from previously confirmed COVID-19 cases. Finally, all samples were obtained for clinical testing or medical examinations and might overrepresent persons with greater healthcare access or concerns for healthcare.

The spread of COVID-19 is difficult to predict. As the virus naturally mutates, the current seroprevalence is changing. Various factors can influence seroprevalence in regions within a country. Prevention and control measures against the spread of SARS-CoV-2 infections differ in each country, as do cultures and local healthcare systems. As new variants emerge, serological studies targeting community-dwelling people must be repeated to understand the changing status of infections in the community.

In conclusion, given the limitations of this serological survey, it is necessary to periodically conduct community-based nationwide surveys. However, despite these limitations, the prevalence of antibodies in the community was not high as of the time of this study; therefore, it seems that Korea’s response to COVID-19 was effective in suppressing the highly contagious virus. With our current experience and knowledge of responding to COVID-19, we hope to develop more effective strategies for the current and future pandemics.

## Figures and Tables

**Table 1.** Study population and registered population distribution by age, sex, and region group

Variables	n (%)	Age, mean±SD	n (%)^1^
Entire capital area	4,942 (100)	47.6±16.7	21,977,556 (100)
Age (yr)			
19-29	898 (18.2)	24.8±3.1	3,927,681 (17.9)
30-39	855 (17.3)	34.8±2.9	3,774,675 (17.2)
40-49	982 (19.9)	45.0±2.9	4,311,425 (19.6)
50-59	967 (19.6)	54.7±2.9	4,279,414 (19.5)
60-69	691 (14.0)	64.0±2.8	3,192,804 (14.5)
≥70	549 (11.1)	76.9±5.4	2,491,557 (11.3)
Sex			
Male	2,454 (49.7)	46.9±16.2	10,844,444 (49.3)
Female	2,488 (50.3)	48.4±17.1	11,133,112 (50.7)
Region			
Gyeonggi Province	2,882 (58.3)	47.6±16.5	11,153,768 (50.8)
Seoul	1,681 (34.0)	47.9±17.1	8,350,242 (38.0)
Incheon	379 (7.7)	47.4±16.5	2,473,546 (11.3)

1 Resident registration population as of February 2021.

**Table 2.** SARS-CoV-2 antibody prevalence by age, region, and sex

Variables		n (%)	Screened positivity (n = 32)^1^			Confirmed positivity (n = 25)^2^			Capital area cumulative incidence (~1/16/2021)^4^
			No. of positive detection	Prevalence	Estimated prevalence^3^	No. of positive detection	Prevalence	Estimated prevalence	
Entire capital area		4,942 (100)	32	0.65 (0.46, 0.91)	0.60 (0.42, 0.85)	25	0.51 (0.34, 0.75)	0.47 (0.31, 0.70)	0.18
Age (yr)									
	19-29	898 (18.2)	3	0.33 (0.11, 0.98)	0.29 (0.09, 0.92)	3	0.33 (0.11, 0.98)	0.29 (0.09, 0.92)	0.17
	30-39	855 (17.3)	7	0.82 (0.40, 1.68)	0.69 (0.32, 1.51)	4	0.47 (0.18, 1.20)	0.40 (0.14, 1.10)	0.16
	40-49	982 (19.9)	2	0.20 (0.06, 0.74)	0.18 (0.05, 0.70)	2	0.20 (0.06, 0.74)	0.18 (0.05, 0.70)	0.15
	50-59	967 (19.6)	8	0.83 (0.42, 1.62)	0.78 (0.39, 1.56)	7	0.72 (0.35, 1.49)	0.69 (0.33, 1.44)	0.19
	60-69	691 (14.0)	6	0.87 (0.40, 1.88)	0.89 (0.41, 1.94)	4	0.58 (0.23, 1.48)	0.61 (0.23, 1.56)	0.23
	≥70	549 (11.1)	6	1.09 (0.50, 2.36)	1.00 (0.45, 2.24)	5	0.91 (0.39, 2.11)	0.79 (0.32, 1.95)	0.23
Sex									
	Male	2,454 (49.7)	15	0.61 (0.37, 1.01)	0.56 (0.34, 0.95)	10	0.41 (0.22, 0.75)	0.38 (0.20, 0.71)	0.18
	Female	2,488 (50.3)	17	0.68 (0.43, 1.09)	0.63 (0.39, 1.03)	15	0.60 (0.37, 0.99)	0.55 (0.33, 0.93)	0.18
Region									
	Gyeonggi Province	2,882 (58.3)	25	0.87 (0.59, 1.28)	0.87 (0.59, 1.28)	20	0.69 (0.45, 1.07)	0.67 (0.45, 1.08)	0.15
	Seoul	1,681 (34.0)	7	0.42 (0.20, 0.86)	0.42 (0.20, 0.86)	5	0.30 (0.13, 0.69)	0.30 (0.13, 0.69)	0.24
	Incheon	379 (7.7)	0	-	-	0	-	-	0.14

Values are presented as % (95% confidence interval). SARS-CoV-2, severe acute respiratory syndrome coronavirus 2; PRNT, plaque reduction neutralization test.

1 Screened positivity was based on the test results using Elecsys anti-SARS-CoV-2 (Roche).

2 Confirmed positivity was based on the serial test results using GenScript or PRNT.

3 Estimation with sampling weight using the population of resident registration.

4 The cumulative incidence rate in the capital area was calculated using the cumulative number of confirmed people aged ≥19 years until January 16, 2021 as a numerator and the resident registration population in February 2021 as the denominator.
